# Influence of Rosa damascena hydrosol on skin flora (contact culture) after hand-rubbing

**DOI:** 10.3205/dgkh000356

**Published:** 2020-09-07

**Authors:** Gulsum Iclal Bayhan, Tulay Gumus, Basak Alan, Isil Kubra Savas, Saliha Aysenur Cam, Elif Ayca Sahin, Seyfullah Oktay Arslan

**Affiliations:** 1 Yildirim Beyazit University, Faculty of Medicine, Yenimahalle Educational and Training Hospital, Ankara, Turkey; 2 Yildirim Beyazit University, Yenimahalle Educational and Training Hospital, Microbiology, Ankara, Turkey; 3 Yildirim Beyazit University, Faculty of Medicine, General Pediatrics, Ankara, Turkey; 4 Yildirim Beyazit University Faculty of Medicine, Pharmacology, Ankara, Turkey

**Keywords:** rosa damascena, hydrosol, hand rubbing, antisepsic efficacy

## Abstract

**Aim and Introduction:** *Rosa damascena* is one of the most well-known species of the Rosaceae family and is widely used in the food and perfume industry. Rose hydrosol is a product which is produced by distillation of rose petals. There is very little research about the antimicrobial effect of rose hydrosol. In this study, we aimed to investigate the antibacterial effect of *Rosa damascena* hydrosol *in vivo*.

**Method:** 45 adult volunteers who were not healthcare workers were included in this study. Exclusion criteria included existing skin disorders or lacerations, pregnancy, presence of nail polish, recent handwashing or use of antiseptic lotion/soap in the last week, and antibiotic use in the last 3 months. At baseline, each subject was asked to rub the fingertips of the dominant hand on a sheep-blood agar plate. The subjects were randomly divided into two groups: one group rubbed their hands with 3 mL of alcohol-based hand antiseptic and the other group with 3 mL of rose hydrosol. Following sample collection, the subjects were asked to rub their hands according to the World Health Organization’s (WHO) “How to Hand Rub” technique. After the hand-rubbing sequence, the hands were allowed to air-dry and fingertip sampling was performed. Culture plates were evaluated by a microbiologist blinded to group assignment. Rose hydrosol was analysed by gas chromatography/mass spectrometry.

**Results:** The main components of rose hydrosol are phenyl ethyl alcohol, beta-citronellol and geraniol. Of the total of 45 participants, 23 were included in rose hydrosol group and 22 in the alcohol-based hand-rub group. The colony counts decreased significantly in the alcohol-based solution group after hand-rubbing, whereas there was no significant reduction in the rose hydrosol group.

**Conclusion:** A number of studies have shown good antimicrobial activity in rose products, especially in rose oil, but we found no antibacterial effect of rose hydrosol after hand-rubbing. However, it must be borne in mind that the amount and types of rose hydrosol components are highly influenced by the given agro-meteorological conditions and technological production methods.

## Zusammenfassung

**Zielsetzung:** *Rosa Damascena* ist eine der berühmtesten Arten der Familie der Rosaceae und wird in breitem Umfang in der Lebensmittel- und Parfümindustrie genutzt. Rosenhydrosol ist ein Produkt, das durch Destillation von Rosenblütenblättern hergestellt wird. Es gibt nur wenige Untersuchungen zur antimikrobiellen Wirkung von Rosenhydrosol. Daher sollte die antibakterielle Wirkung von *Rosa damascena* Hydrosol *in vivo* untersucht werden.

**Methode:** 45 freiwillige Erwachsene, die nicht im Gesundheitswesen tätig sind, wurden in die Studie eingeschlossen. Ausschlusskriterien waren bestehende Hauterkrankung oder Hautverletzung, Schwangerschaft, Nagellack, vor kurzem durchgeführte Händewaschung, Benutzung antiseptischer Lotion/Seife in der letzten Woche und die Einnahme von Antibiotika in den letzten 3 Monaten. Zu Versuchsbeginn wurde jeder Proband gebeten, die Fingerspitzen der dominanten Hand auf der Schafblutagar aufzudrücken. Die Versuchspersonen wurden randomisiert zwei Gruppen zugeordnet. Eine Gruppe rieb 3 ml eines alkoholbasierten Händedesinfektionsmittels, die andere Gruppe 3 ml Rosenhydrosol gemäß der WHO-Empfehlung „How to Hand Rub“ ein. Nach dem Einreiben wurden die Hände an der Luft an der Luft getrocknet und erneut Proben von den Fingerspitzen entnommen. Die Anzahl KbE wurden von einem Mikrobiologen verblindet ausgewertet. Das Rosenhydrosol wurde mittels Gaschromatographie/Massenspektrometrie analysiert.

**Ergebnisse:** Die Hauptbestandteile von Rosenhydrosol waren Phenylethylalkohol, Beta-Citronellol und Geraniol. Von den 45 Teilnehmern wurden 23 in die Rosenhydrosol-Gruppe und 22 in die Gruppe der alkoholbasierten Handeinreibungen aufgenommen. Die Koloniezahlen nahmen nach Händedesinfektion signifikant ab, während es in der Gruppe mit Rosenhydrosol keine signifikante Reduktion gab.

**Schlussfolgerung:** Einige Studien haben eine gute antimikrobielle Aktivität in Rosenprodukten und speziell in Rosenöl gezeigt. Bei Einreiben in die Hand konnten wir keine antibakterielle Wirkung von Rosenhydrosol nachweisen. Es ist jedoch zu berücksichtigen, dass die Menge und die Art der Rosenhydrosol-Komponenten stark von den jeweiligen agro-meteorologischen Bedingungen und den technologischen Produktionsmethoden beeinflusst werden.

## Introduction

*Rosa damascena* is one of the most well-known of the approximately 10,000 species of the Rosaceae family and is widely used in the food and perfume industry. The four main products of *Rosa damascena* are rose oil, rose hydrosol (rose water), rose petal extract and dried rose petals. Hypnotic, analgesic, laxative, anti-diabetic, anti-inflammatory and antioxidant effects of *Rosa damascena* have been reported [1], [2]. Its antimicrobial effect has also been the subject of research, but there are only a few related articles, mostly involving rose oil and rose petal extracts. Rose oil and alcohol or aqueous extracts of rose petal have been reported to possess various antimicrobial activities [3], [4], [5], [6]. Rose hydrosol has a pleasant, refreshing aroma, and is generally used for skin care due to its hydrating and anti-inflammatory effects. 

However, there is very little research about the antimicrobial effect of rose hydrosol, a product produced by the distillation of rose petals, which is also known as “rose water” [2], [7]. Thus, we aimed to investigate the *in vivo* antibacterial effect of *Rosa damascena* hydrosol in this study.

## Methods

### Participants and sample collection

The study was approved by the ethics committee of the University Faculty of Medicine, Ankara (registration number 2019/02/06). A total of 45 adult volunteers who were not healthcare workers were included in this study. The sample size was calculated as 16 with a 95% confidence interval and 80% power using the OpenEpi (https: //www.openepi.com/SampleSize/SSPropor.Htm) programme [8]. We included 40% more than the calculated value. Exclusion criteria included existing skin disorders or lacerations, pregnancy, presence of nail polish, recent hand washing or use of antiseptic lotion/soap use in the last week, and antibiotic use in the last 3 months. Gender, age and the dominant hand were recorded. Informed consent was obtained from all participants. 

At baseline, each subject was asked to rub the fingertips of the dominant hand on sheep-blood agar plate. Following sample collection, the subjects were asked to rub their hands according to the World Health Organization’s (WHO) “How to Hand Rub” technique [9]. The subjects were randomly divided into two groups: one group rubbed their hand with 3 mL of alcohol-based hand antiseptic and the other group with 3 mL of rose hydrosol. After the hand-rubbing sequence, the hands were allowed to air-dry and fingertip sampling was performed again. 

The commercially available product (MANOCHOL™ EP-70) used as the alcohol-based hand rub contained 70% ethanol. The *Rosa damascena* hydrosol used was a commercial product produced according to the national standard of the Turkish Standards Institution (TSE, “Rose Water Monograph” TS 5555, 1988). 

### Bacteriological study

Each plate was immediately taken to the microbiology department and incubated at 37°C for 48–72 hours. The culture plates were evaluated by a microbiologist blind to group assignment. Colony-forming units (CFU) were counted when fewer than 100 CFU were present and approximated otherwise; the results were divided into 4 groups: (+) (0–20 CFU), (++) (21–50 CFU), +++ (51–99 CFU), and (++++) (≥100 CFU).

### Gas chromatography/mass spectrometry (GC-MS) of rose water

#### Extraction procedure

Extraction of the volatile components of rose water was performed as described in the literature [10]. Briefly, 100 mL of rose water was placed in a glass flask and 2 g of NaCl was added. After the addition of 2.5 mL of ethyl acetate, the flask was placed in an ultrasonic bath and sonicated for 30 min at room temperature. The ethyl acetate phase was collected and rose water was re-extracted. The ethyl acetate phase was concentrated under nitrogen and then transferred to a vial for GC-MS analysis.

## Results

The 45 subjects were divided into an alcohol-based solution group with 22 subjects and a rose water group with 23 subjects. Male/female ratio (8/14 in alcohol-based solution group and 4/19 in rose water group p=0.189) and age were similar between two groups (p=0.42). 

The colony counts before and after the hand-rubbing are presented in Table 1 (Tab. 1). The colony counts decreased significantly in the alcohol-based solution group after hand-rubbing, whereas there was no significant reduction in the rose water group. 

### Pharmacological analysis of rose hydrosol

**Figure 1:**
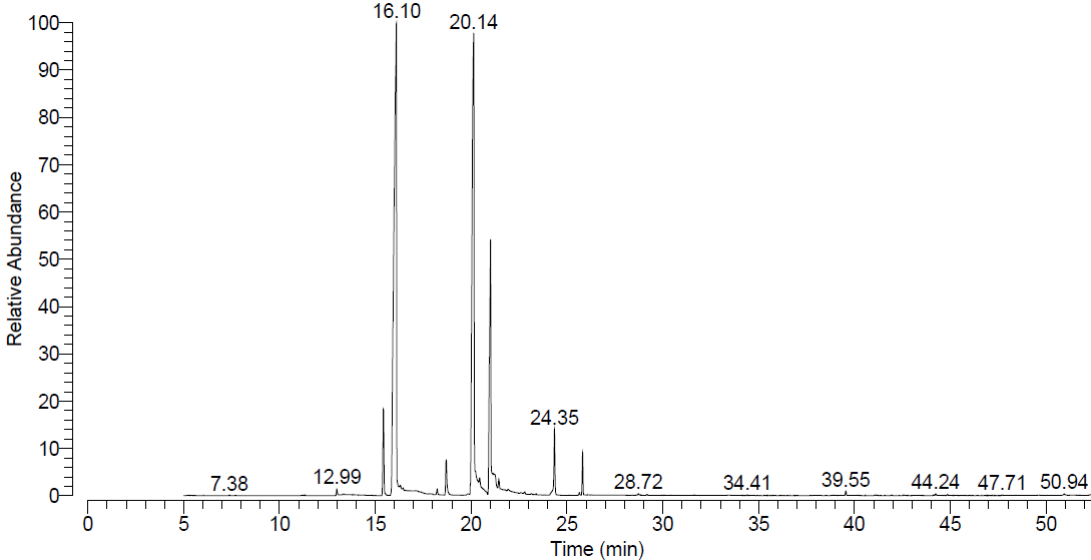
Chemical compounds detected in rose water with GC/MS (RT: retention time)

Figure 1 (Fig. 1) shows the ion chromatogram of the GC-MS analysis. The main components of the ethyl acetate extract of rose water are phenyl ethyl alcohol (45.4%), beta-citronellol (34.12) and geraniol (12.16). Other components are eugenol (2.18%), linalool (2.11%), alpha-terpineol (1.31%), methyl eugenol (1.26%), benzyl alcohol (0.44%), eicosane (0.11%), terpinen 4-ol (0.08%), and nerol (0.05%) (Table 2 (Tab. 2)).

## Discussion

Rose hydrosol is produced by the distillation method. Distillation is performed at high temperatures (100°C) and partial pasteurisation is therefore achieved. Microbiological analyses of rose water have shown that the microbiological load is very low, and that the microbiological load in the same sample was stable after 1.5 years [11]. This may indicate a natural resistance to bacterial colonisation. There was no or very little change in the amount of the major constituents citronellol, geraniol and nerol in the rose water samples during storage for one year, while the amount of phenylethyl alcohol increased and there was some change in the minor components [12]. The rose hydrosol component can therefore stay relatively stable during the storage period. 

Rose oil is the best-investigated product of *Rosa damascene* in regard to antimicrobial activity, but conflicting results have been reported. One study has found that *Rosa damascene* essential oil has antimicrobial activity against *S. aureus* but not against *E. coli* and *P. aeruginosa *[13]. Other authors reported that rose oil has an antibacterial effect against *S. aureus*, *P. aeruginosa*, and *E. coli* [11]. A further product of *Rosa damascene* is rose petal extracts, the antibacterial activity of which has been studied. The aqueous extract, and the methanol, ethanol and butanol extracts of *Rosa damascene* have been shown to possess various kinds of antimicrobial activity [2], [14], [15], [16]. The antimicrobial activity differs among different types of extracts, possibly due to the different chemicals found with different extraction methods that could affect the bacterial death rate. The antimicrobial activity has also been shown to vary depending on the extract concentration [2], [14], [15], [16].

The presence of an antibacterial effect in rose oil and extract does not mean that rose hydrosol is also expected to have such an effect, due to different chemical compositions. A study from Turkey found that *Rosa damascane* hydrosol has the four major components geraniol (30.7%), citrenellol (29.4%), phenylethyl alcohol (23.7%) and nerol (16.1%) that are also found in rose oil and rose abstract, whereas the hydrosol contains only a few phenolic components at much lower amounts than in rose oil and rose abstract. The same study found that rose oil had an antibacterial effect against *P. aeruginosa*, *E. coli*, and *S. aureus* whereas rose hydrosol did not, perhaps because the antibacterial activity is mainly due to the phenolic contents [2]. A different study showed that rose hydrosol does not have antibacterial activity against *Listeria* species [7]. As mentioned above, we found that the major components of rose hydrosol were phenylethyl alcohol, citronellol and geraniol, with only little phenolic content. The colony count decreased significantly following alcohol-based hand-rubbing but did not show significant change with rose hydrosol, i.e. it did not show signs of antibacterial activity. 

Geraniol, citronellol and nerol have been reported to possess more potent antibacterial activity separately than as a mixture [13]. Geraniol, an acyclic isoprenoid monoterpene, is one of the main conponents of rose water and has been shown to have various pharmacological effects, including antibacterial activity [17]. Eugenol is a phenolic monoterpene and its antibacterial effect has been demonstrated against various bacterial species [18], [19], [20], [21]. Alpha-terpinol also has an antibacterial effect [22]. A recent study has reported that beta-citronellol has anti-fungal activity against *Candida albicans* [23]. Because rose hydrosol contains all of these components, the absence of antibacterial activity may therefore be due to antagonism between these alcoholic contents. 

A study comparing the antimicrobial activity of five types of *Rosa damascene* oil obtained from various countries has shown that the effect of the rose oil types was different [6]. It is possible that the difference is due to the agro-meteorological conditions and industrial production methods used. The chemical composition of *Rosa damascene* varies depending on the flower development stage (half bloom, full bloom, and bud stage), climatic conditions, altitude, and genetic variability [20]. This difference may also result in different chemical compositions in different rose hydrosol products and therefore potential differences in antibacterial effect. 

In conclusion, a number of studies have shown good antimicrobial activity for rose products, especially rose oil, but we found no antimicrobial effect of rose hydrosol. However, one must consider that the amount and types of the rose hydrosol components are highly influenced by the relevant agro-meteorological conditions and industrial production methods.

## Notes

### Competing interests

The authors declare that they have no competing interests.

## Figures and Tables

**Table 1:**
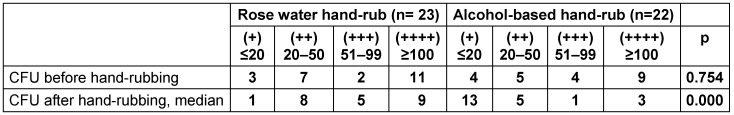
Comparison of the colony count before and after hand-rubbing with alcoholic hand-rub and rose water

**Table 2:**
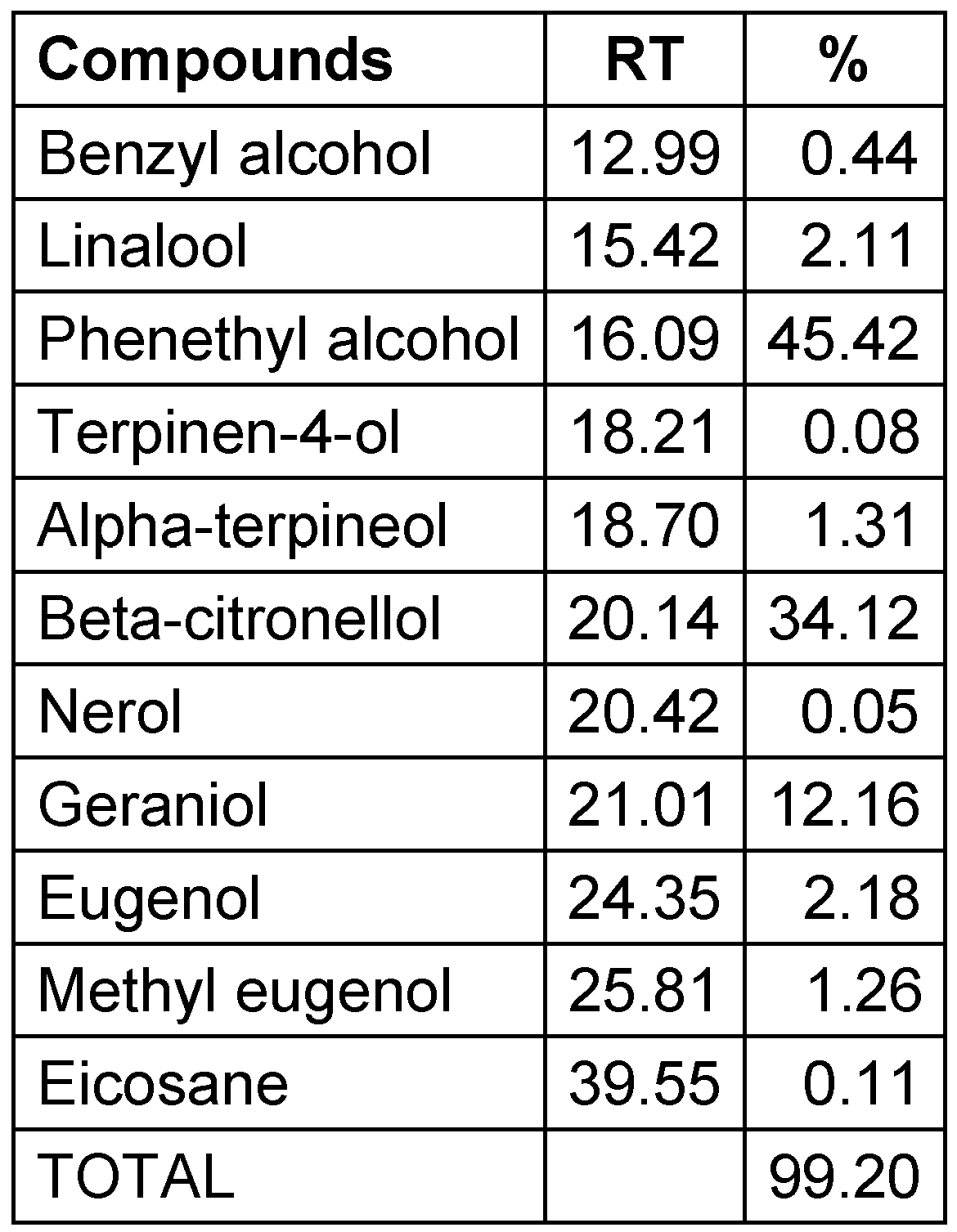
Chemical compounds detected in rose water with GC/MS (RT: Retention time)
